# A newly-recorded genus, *Lasiocallimerus* Corporaal, 1939 (Coleoptera, Cleridae) from China, with description of a new species

**DOI:** 10.3897/BDJ.13.e169936

**Published:** 2025-10-23

**Authors:** Zichao Chai, Jiří Kolibáč, Haoyu Liu, Yali Yu, Jihuan Zheng

**Affiliations:** 1 The Key Laboratory of Zoological Systematics and Application, College of Life Science, Institute of Life Science and Green Development, Hebei University, Baoding, China The Key Laboratory of Zoological Systematics and Application, College of Life Science, Institute of Life Science and Green Development, Hebei University Baoding China; 2 Guangdong Key Laboratory of Animal Conservation and Resource Utilization, Institute of Zoology, Guangdong Academy of Sciences, Guangzhou, China Guangdong Key Laboratory of Animal Conservation and Resource Utilization, Institute of Zoology, Guangdong Academy of Sciences Guangzhou China; 3 Moravian Museum; Department of Entomology, Brno, Czech Republic Moravian Museum; Department of Entomology Brno Czech Republic

**Keywords:** Cleroidea, Hydnocerini, taxonomy, new faunistic record, checkered beetles

## Abstract

**Background:**

The genus *Lasiocallimerus* Corporaal, 1939 is a small genus belonging to the tribe Hydnocerini in the subfamily Clerinae (Coleoptera, Cleridae), with three known species from Southeast Asia.

**New information:**

This genus is recorded from China for the first time. A new species, *Lasiocallimerus wangi* sp. nov., is described and illustrated from Yunnan Province and *L. dembatkoi* Kolibáč, 1998 is newly recorded from Yunnan and Guangxi Provinces of China. A key to the identification of all *Lasiocallimerus* species is provided.

## Introduction

The genus *Lasiocallimerus* Corporaal was erected by Corporaal (1939) for the type species, *Lasiocallimerus vestitus* Corporaal, 1939, which differs from most species of the genus *Callimerus* in having “Body robust, large, about 5-8 mm. Eyes extremely protuberant, contour of eyes exceeds width of elytra. Antennae 10-segmented, only terminal segment clubbed (*Callimerus* with terminal 3-segmented clubbed). Labrum angulate, without armature on pharyngeal side. Empodium without setae” (Kolibáč 1998). Three *Lasiocallimerus* species have been described, *L. vestitus* Corporaal, 1939, *L. pacholickyi* Kolibáč, 1998 and *L. dembatkoi* Kolibáč, 1998, all distributed in Southeast Asia (Corporaal 1939, Kolibáč 1998).

The genus *Lasiocallimerus* belongs to Hydnocerini in subfamily Clerinae, since Bartlett (2021) synonymised Hydnocerinae with Clerinae and lowered Hydnocerinae to tribe. Afterwards, two new genera, *Tarsobaenus* Leavengood et al., 2022 from Costa Rica and *Neohydnocera* Leavengood, 2025, were described (Leavengood Jr et al. 2022, Leavengood Jr 2025). With the inclusion of genus *Cephaloclerus* Kuvwet, resurrected from *Phyllobaenus* Dejean, 1877 (Leavengood Jr 2025), the tribe Hydnocerini comprises 19 genera, of which 10, including *Lasiocallimerus*, belong to Hydnocerina.

Three genera, *Callimerus* Gorham,1876, *Neohydnus* Gorham, 1892 and *Emmepus* Motschulsky, 1845 of Hydnocerini in China were recored (Yang 2012). In this study, a new species, *L. wangi* sp. nov., is described and *L. dembatkoi* Kolibáč, 1998 is newly recorded from Yunnan and Guangxi Provinces, representing the first record of the genus *Lasiocallimerus* in China.

## Materials and methods

### Specimens examined

The morphological characters were examined with an Olympus SZ61 microscope. Habitus images were taken using a Canon EOS R8 connected with Mitutoyo macro lens M-Plan Apo 5× and 10× through a Novoflex Micro-Tube and a new species was photographed with a Canon 5DSR/Nikon SMZ25 digital camera. Whole female abdomens were removed from the body with fine forceps, treated with 10% potassium hydroxide (KOH) solution at room temperature for 12–16 hours. Female reproductive organs were dissected, photographed in 70% ethanol, then mounted on a plastic microslide in Euparal, which was pinned below specimens. A cable shutter release was used to prevent the camera from shaking. To obtain the full depth of focus, all images were stacked using HELICON FOCUS 7 and the resulting output was edited with Adobe Photoshop 2022.

In this paper, we follow the classification system of Bartlett (2021).

The material in this study is deposited in the following institutions: Institute of Zoology, Chinese Academy of Sciences, Beijing, China (IZAS); Institute of Zoology, Guangdong Academy of Sciences (IZGAS); Naturalis Biodiversity Center, Amsterdam, Netherlands (NBC); The collection of J. Kolibáč in Moravian Museum (Coll. MMJK).

## Taxon treatments

### Lasiocallimerus

Corporaal, 1939

993CC8ED-416D-503F-8D97-C3F06BFDD051

Lasiocallimerus vestitus Corporaal, 1939

#### Diagnosis

Body robust, 5–8 mm. Head including eyes slightly wider than pronotum, punctured finely and evenly, with dense white setae and long erect pubescence; apical margin of labrum emarginate at middle, maxillary terminal palpomere digitiform, labial terminal palpomere triangular; gular sutures parallel, post gular plate wide; antennae 10-segmented, relatively short, only terminal segment clubbed; eyes large, very slightly emarginated near antennal insertions, finely granulate. Tibial spur formula 2–2–2; tarsi compact, first tarsomeres of all pairs of legs minute; tarsal pulvillar formula 4–4–4, first indistinct, remaining distinct; claws with denticle.

Compared with the genera of Hydnocerini in China, *Lasiocallimerus* differs from *Callimerus* obviously by the only terminal segment clubbed, whereas *Callimerus* terminal 3-segmented clubbed; markedly differs from *Emmepus* by elytra enough to completely cover the abdomen (*Emmepus* elytra strongly shortened). *Lasiocallimerus* is most similar to *Neohydnus*, but can be separated by its more robust body, distinct colour patterns and with extremely protuberant eyes (Kolibáč 1998).

#### Distribution

China (Yunnan, Guangxi), India (Assam), Thailand, Laos, Indonesia (Java), Malaysia (Sabah).

### Lasiocallimerus vestitus

Corporaal, 1939

48D41335-B5CD-513D-B851-FBF1E448782A

#### Materials

- **Type status:** Holotype. **Occurrence:** catalogNumber: ZMA. INS. COLE. 1756. 1.; recordedBy: Mrs. E. Walsh; individualCount: 1; sex: female; lifeStage: adult; occurrenceID: AAC7A30D-0A2F-585C-9DDE-97D9CFE22E3F; **Taxon:** scientificName: *Lasiocallimerus vestitus*; **Location:** country: Indonesia; stateProvince: Java; locality: Zuid Bantam; georeferenceProtocol: label; **Event:** eventDate: 15-8/15-9-1934; **Record Level:** language: en; institutionCode: NBC; collectionCode: Insects; basisOfRecord: PreservedSpecimen

#### Diagnosis

**Figure 1.**
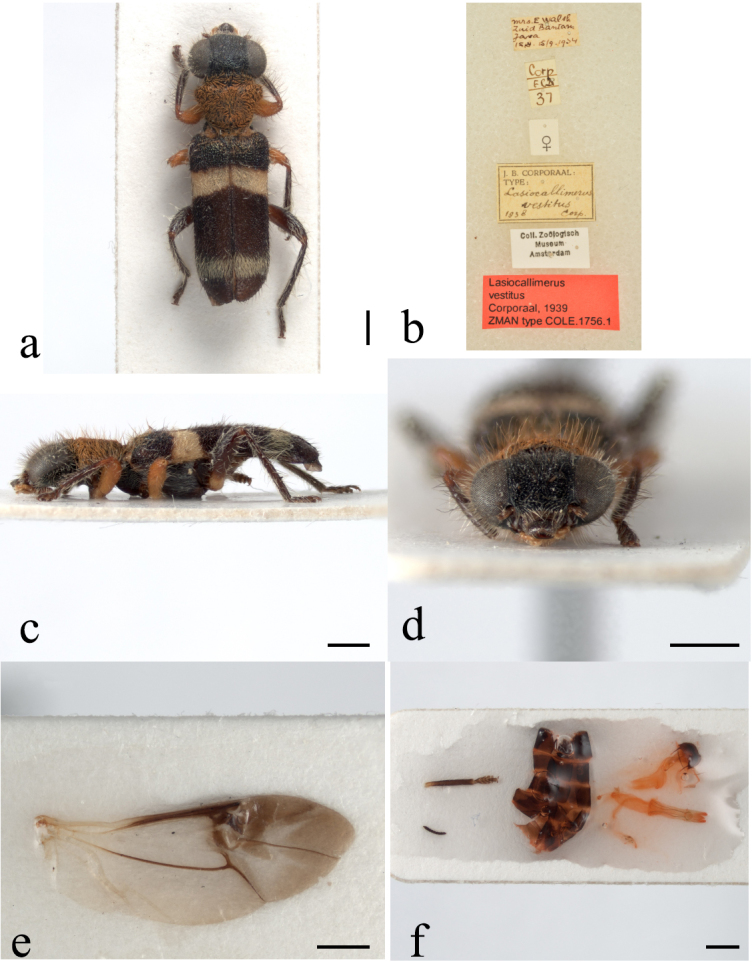
*Lasiocallimerus vestitus* holotype (Registration number: No. ZMA. INS. COLE. 1756. 1). **a** habitus in dorsal view; **b** labels; **c** habitus in lateral view; **d** head in frontal view; **e** wing; **f** middle leg, abdomen and female internal copulatory organs. Scale bars = 1 mm.

This species is markedly differs from other three *Lasiocallimerus* species in pronotum covered with brick-red setar. Although it is similar to *L. dembatkoi* in sharing a white-yellow transverse band on elytra basal third, it additionally exhibits a darker band at the elytral apex (Fig. 1).

#### Distribution

Thailand, Laos, Indonesia(Java).

### Lasiocallimerus dembatkoi

Kolibáč, 1998

145DF25D-A864-5F5E-A468-D801E4CAB703

#### Materials

- **Type status:** Holotype. **Occurrence:** recordedBy: Pacholatko & Dembicky; individualCount: 1; sex: female; lifeStage: adult; occurrenceID: 268035F0-4F4A-5003-A1E1-D6E93ECBB41A; **Taxon:** scientificName: *Lasiocallimerus dembatkoi*; **Location:** country: Thai; locality: Soppong Pai; verbatimElevation: 1800; georeferenceProtocol: label; **Event:** eventDate: 15-8/15-9-1934; **Record Level:** language: en; institutionCode: Coll. MMJK; collectionCode: Insects; basisOfRecord: PreservedSpecimen
- **Type status:** Other material. **Occurrence:** recordedBy: Mangyi Nong; individualCount: 1; sex: female; lifeStage: adult; occurrenceID: 9EDDAABE-5889-5C09-8F90-FA82E8EAC9C9; **Taxon:** scientificName: *Lasiocallimerus dembatkoi*; **Location:** country: China; locality: Yunnan Province Xishuangbanna Autonomous Prefecture; georeferenceProtocol: label; **Event:** eventDate: 15/30-IV-2024; **Record Level:** language: en; institutionCode: IZGAS; collectionCode: Insects; basisOfRecord: PreservedSpecimen
- **Type status:** Other material. **Occurrence:** recordedBy: Hui Xiao; individualCount: 1; sex: female; lifeStage: adult; occurrenceID: FCA32F19-1C37-5094-B69A-E92B6AB081C0; **Taxon:** scientificName: *Lasiocallimerus dembatkoi*; **Location:** country: China; locality: Guangxi Province, Jinxiu Yao Autonomous County, Jinzhong Road; verbatimElevation: 1100; georeferenceProtocol: label; **Event:** eventDate: 12-V-1999; **Record Level:** language: en; institutionCode: IZAS; collectionCode: Insects; basisOfRecord: PreservedSpecimen

#### Diagnosis

**Figure 2.**
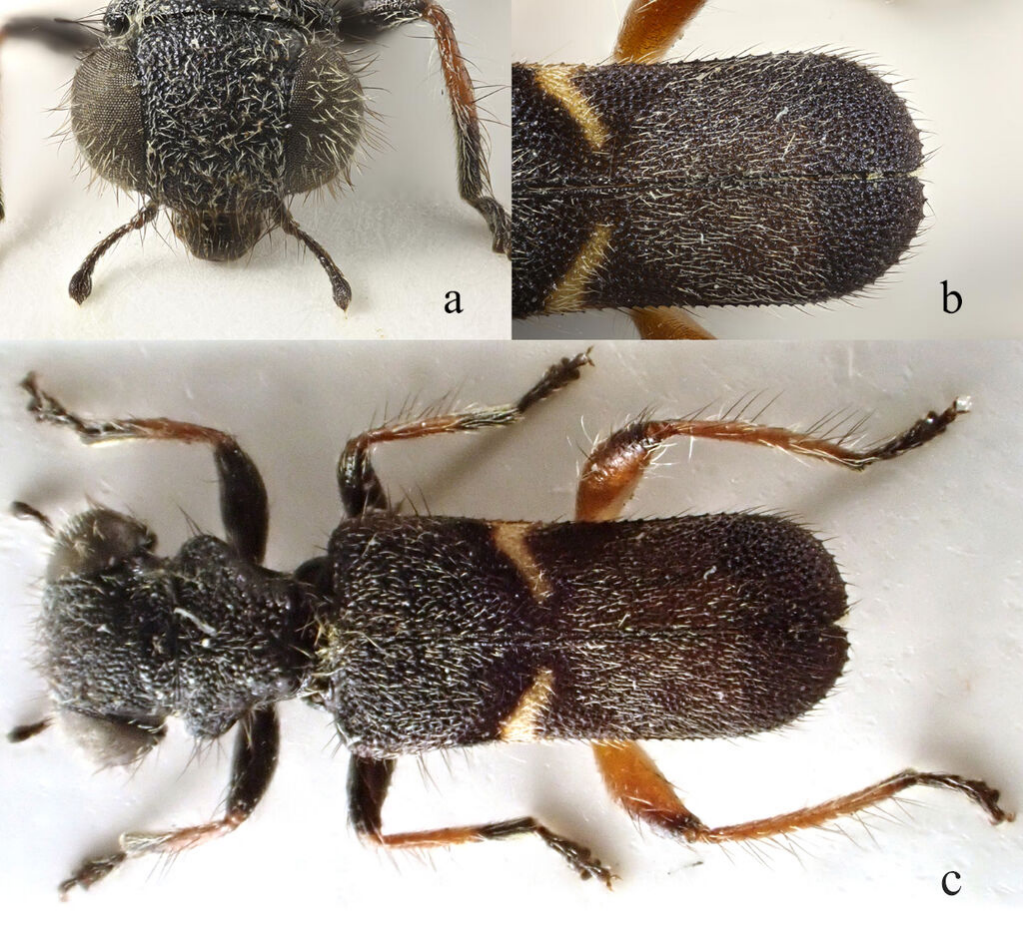
*Lasiocallimerus dembatkoi* holotype. **a** head in frontal view; **b** elytra; **c** habitus in dorsal view.

**Figure 3.**
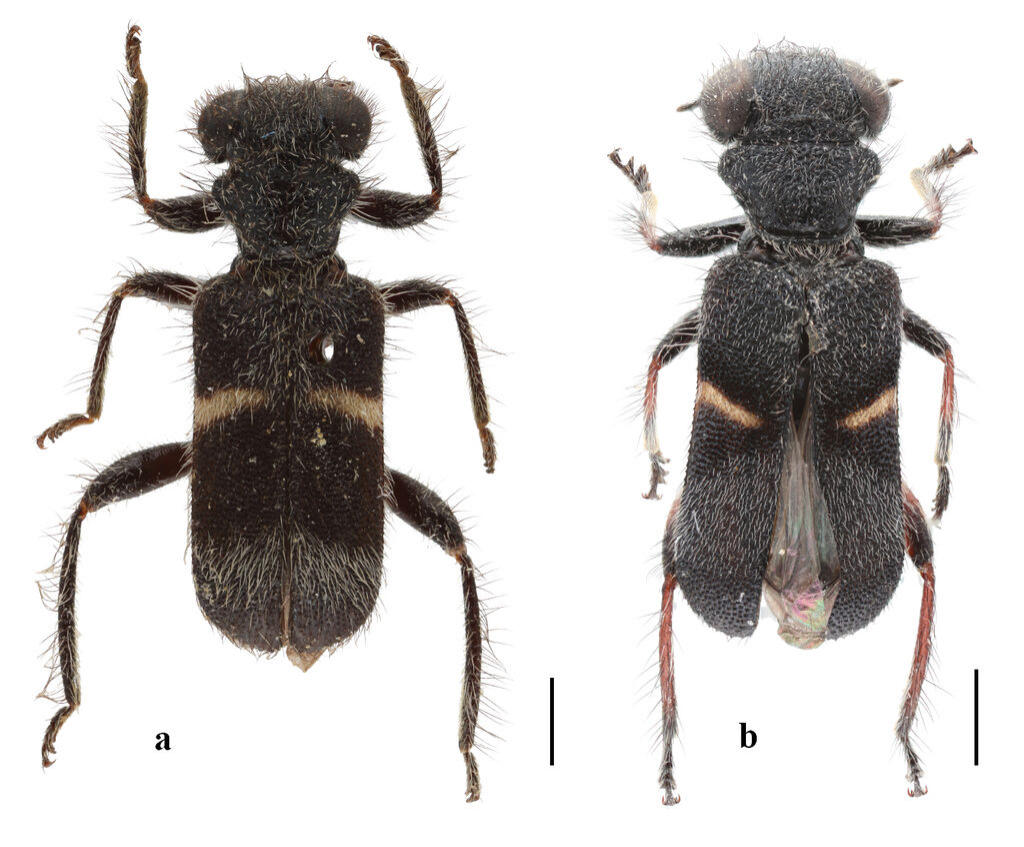
*Lasiocallimerus dembatkoi* from China, habitus in dorsal view. **a** Yunnan; **b** Guangxi). Scale bars = 1 mm.

It can be differentiated from other three species by head with white setae and elytra with a single yellow transverse band (Fig. 2, Fig. 3).

#### Distribution

China (Yunnan and Guangxi, new record), Thailand, India (Assam) and Malaysia (Sabah).

#### Notes

The specimens from Yunnan and Guangxi were identified as *L. dembatkoi*, based on the external morphology, which is newly recorded from China. It has been previously recorded from Southeast Asia (Thailand, India, Malaysia). The colour of the legs in this species is variable. A specimen from Guangxi has legs that are entirely black (Fig. 3a) and the specimen from Yunnan has legs that are orange-yellow with the front and middle femora and the distal part of the tibia black (Fig. 3b).

### Lasiocallimerus pacholickyi

Kolibáč, 1998

AA746DB4-8BD1-546F-BCA4-83BBD2C1C91B

#### Materials

- **Type status:** Holotype. **Occurrence:** recordedBy: Pacholatko & Dembicky; individualCount: 1; sex: male; lifeStage: adult; occurrenceID: B495F22D-B9F5-56E9-9D6F-DD5E00D38B4C; **Taxon:** scientificName: *Lasiocallimerus pacholickyi*; **Location:** country: Thai; locality: Soppong Pai; verbatimElevation: 1800; georeferenceProtocol: label; **Event:** eventDate: 15-8/15-9-1934; **Record Level:** language: en; institutionCode: Coll. MMJK; collectionCode: Insects; basisOfRecord: PreservedSpecimen

#### Diagnosis

**Figure 4.**
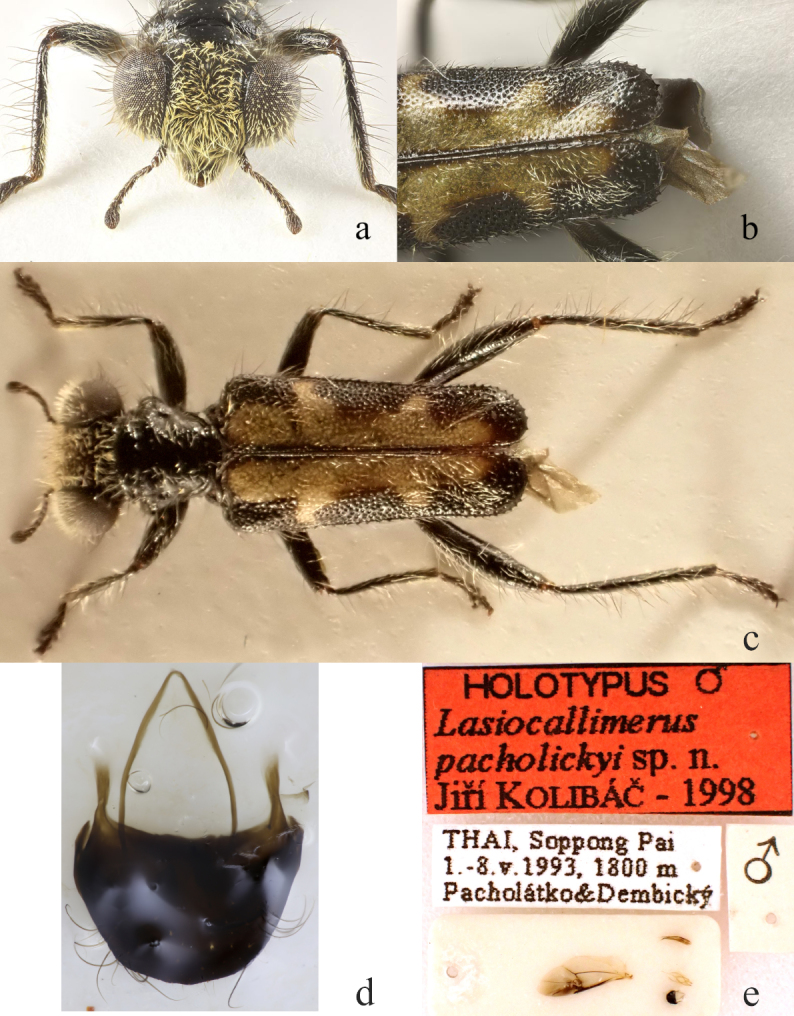
*Lasiocallimerus pacholickyi* holotype. **a** head in frontal view; **b** elytra; **c** habitus in dorsal view; **d** Tergite VIII; **e** labels, wing and male genitalia.

The species can be distinguished from its congeners by elytra with large and complex yellowish pattern along elytral suture reaching nearly from base to apex (Fig. 4).

#### Distribution

Thailand (collected together with *L. dembatkoi*).

### Lasiocallimerus wangi sp. nov.

0CA443E2-E6D7-514A-A877-9004C160BDAC

C480015D-E0FF-4EBE-AAFF-A3C3E9984C72

#### Materials

- **Type status:** Holotype. **Occurrence:** catalogNumber: IOZ(E)1125075; recordedBy: Shuyong WANG; individualCount: 1; sex: female; lifeStage: adult; occurrenceID: CB0D5D5F-605F-537E-B09E-DF548376FE41; **Taxon:** scientificName: *Lasiocallimerus wangi*; **Location:** country: China; stateProvince: Yunnan; locality: Kongming Mountain, Xishuangbanna; verbatimElevation: 2300 m; georeferenceProtocol: label; **Event:** eventDate: 25-XI-1959; **Record Level:** language: cn; institutionCode: IZAS; collectionCode: Insects; basisOfRecord: PreservedSpecimen

#### Description

**Figure 5.**
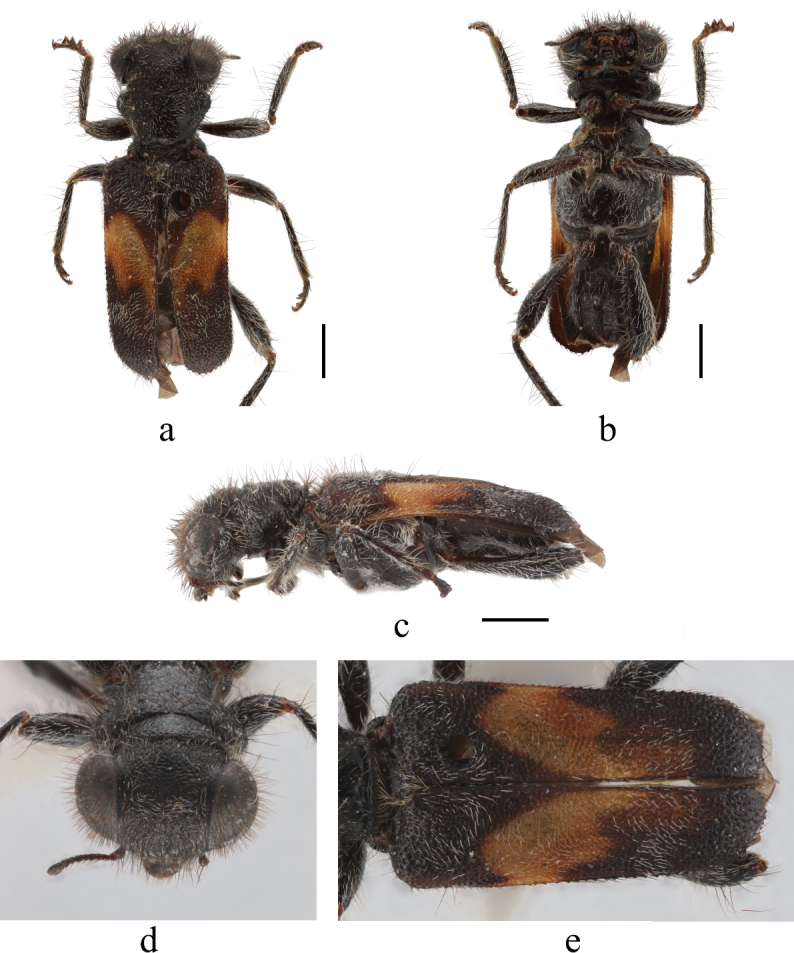
Habitus of *Lasiocallimerus wangi* sp. nov., holotype, female. **a** habitus in dorsal view; **b** ditto, ventral view; **c** ditto, lateral view; **d** head in frontal view; **e** elytra. Scale bars = 1 mm.

Female (Fig. 5). Length 6.5 mm, width 2.35 mm. Head, pronotum, scutellum, ventral side of body black; labrum, labium, labial palpi, maxillary palpi, femora, tibiae, tarsi and claws black-brown; elytra with an irregular orange yellow patch extending from the basal fourth to the apical fourth, with a V-shaped boundary along the suture, blurred posteriorly, the remainder black; body with dense black setae and white pubescence.

Head including eyes slightly wider than pronotum, punctured finely and evenly, with dense white setae and long black erect pubescence; apical margin of labrum emarginate at middle, maxillary terminal palpomere digitiform, labial terminal palpomere triangular; gular sutures parallel, post gular plate wide; antennae 10-segmented, relatively short, terminal segment club; eyes large, very slightly emarginated near antennal insertions, finely granulate (Fig. 5d).

Prothorax wider than long, widest at anterior margin; pronotum distinctly extended behind subapical depression, with dense white setae and long black erect hairs, punctured finely and evenly; sub-basal transverse depressions distinct; scutellum sub-circular, with short white hairs; front and middle coxal cavities open wide.

Elytra almost parallel, twice as long as wide, wider than pronotum, punctured finely and evenly, with short white pubescence (Fig. 5e).

Legs with dense long white hairs; femora long and strong; tibiae with longitudinal carina on dorsal and ventral surface; tibial spur formula 2–2–2; tarsi compact, first tarsomeres of all pairs of legs minute; tarsal pulvillar formula 4–4–4, first indistinct, remaining distinct; claws with denticle.

**Figure 6.**
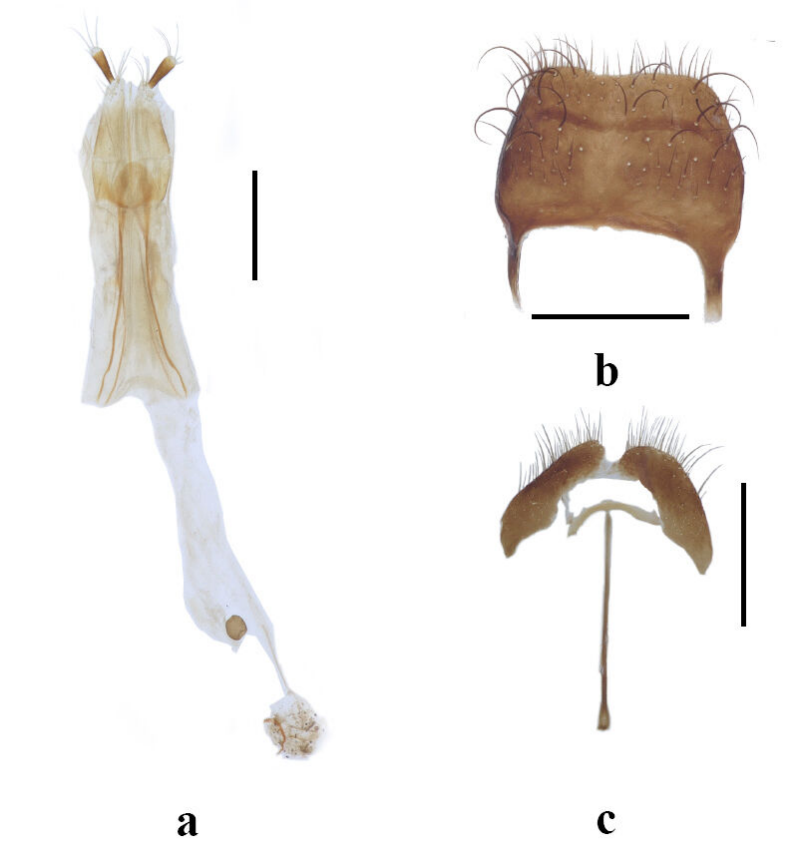
Female genitalia of *Lasiocallimerus wangi* sp. nov. **a** Ovipositor; **b** Tergite VIII; **c** Sternite VIII. Scale bars = 0.5 mm.

Abdomen with six ventrites, anterior margins of ventrites I–IV parallel-side; abdomen completely covered by elytra; with short, brown sparse reclinate setae; ventrites I–V membranously connected; ventrite V flat; lamina of ovipositor bilobed apically (Fig. 6a), tergite VIII and sternite VIII as shown in Fig. 6 (Fig. 6b and c).

Male: Unknown.

#### Diagnosis

The new species differs from others in its complex yellow-orange elytral pattern (large patch) which is situated from the second and third quarter of each elytron (Fig. 5e).

#### Etymology

The new species is named after Mr. Shuyong WANG, the collector of the new species.

#### Distribution

China (Yunnan).

#### Notes

The leg colouration of *L. dembatkoi* and *L. pacholickyi* is variable, with legs entirely black or a combination of black and yellow. As only a single specimen of *L. wangi* sp. nov. is available, it remains unknown whether it also exhibits variable leg colouration.

## Identification Keys

### Key to the species of *Lasiocallimerus*

**Table:** 

1	Pronotum with brick-red setae, elytra dark with two white-yellow transverse bands, front and middle femora reddish-brown (Fig. 1)	*L. vestitus*
–	Pronotum with black or white setae, elytra dark with different pattern	2
2	Elytra black-blue with single yellow transverse band at middle (Fig. 2, Fig. 3)	*L. dembatkoi*
–	Elytra with complex colouration pattern	3
3	Elytra with large and complex yellowish pattern along elytral suture reaching nearly from base to apex, legs entirely black or only base of femora black, remainder yellow (Fig. 4)	*L. pacholickyi*
–	Elytra with a large orange yellow patch in the 2^nd^ to 3^rd^ quarter of length, legs black-brown (Fig. 5)	*L. wangi* sp. nov.

**Updated and modified from Kolibáč (1998)**

## Discussion

**Figure 7.**
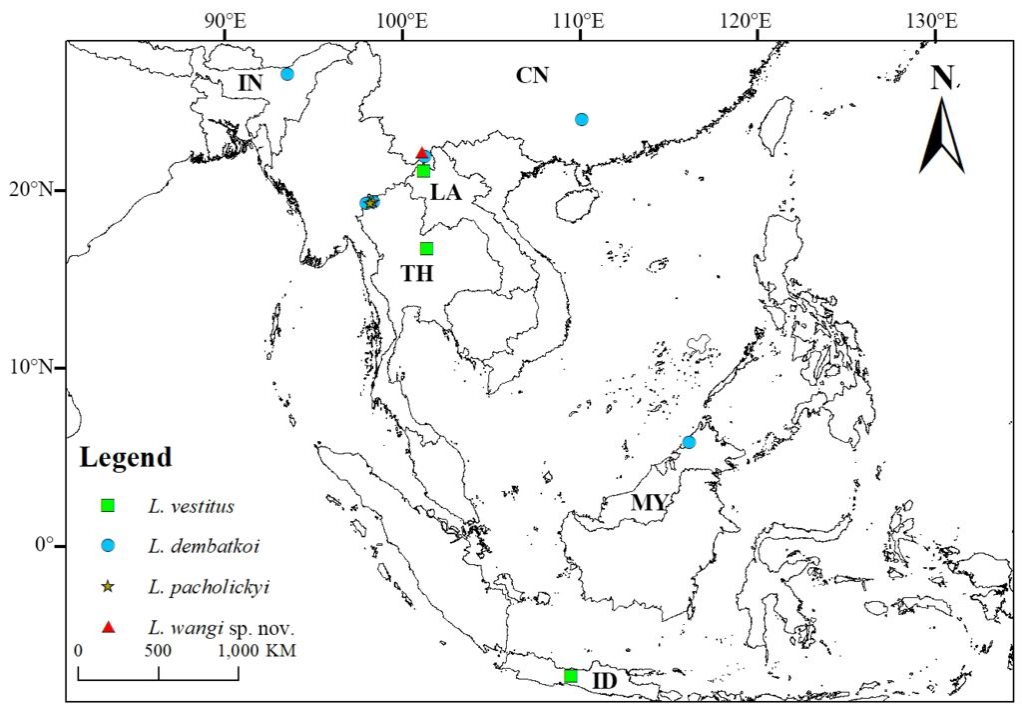
Distribution of the genus *Lasiocallimerus*.

This genus was initially found in Indonesia (Java), with the type species *Lasiocallimerus vestitus* Corporaal, 1939. Later discoveries included *L. pacholickyi* from Thailand and *L. dembatkoi* from Thailand, India (Assam) and Malaysia (Sabah). The new species described herein comes from Yunnan, while *L. dembatkoi* is newly recorded from Yunnan and Guangxi, southern China. This distribution pattern indicates that the genus exhibits a tropical-subtropical regional distribution centred in Southeast Asia (Fig. 7). Further study may discover additional new species across Southeast Asia. Detailed studies of male genitalia are recommended for future research to clarify the phylogenetic relationships within the genus.

## Supplementary Material

XML Treatment for Lasiocallimerus

XML Treatment for Lasiocallimerus vestitus

XML Treatment for Lasiocallimerus dembatkoi

XML Treatment for Lasiocallimerus pacholickyi

XML Treatment for Lasiocallimerus wangi
